# Targeting Insulin-Degrading Enzyme in Insulin Clearance

**DOI:** 10.3390/ijms22052235

**Published:** 2021-02-24

**Authors:** Malcolm A. Leissring, Carlos M. González-Casimiro, Beatriz Merino, Caitlin N. Suire, Germán Perdomo

**Affiliations:** 1 Institute for Memory Impairments and Neurological Disorders, University of California, Irvine (UCI MIND), Irvine, CA 92697-4545, USA; 2 Instituto de Biología y Genética Molecular (University of Valladolid-CSIC), 47003 Valladolid, Spain; carlosmanuel.gonzalez.casimiro@uva.es (C.M.G.-C.); bmerino@ibgm.uva.es (B.M.); 3 Department of Biomedical Sciences, Florida State University, Tallahassee, FL 32306-4300, USA; caitlin.suire@med.fsu.edu

**Keywords:** insulin-degrading enzyme, insulin clearance, liver, pharmacological inhibitors, insulin resistance

## Abstract

Hepatic insulin clearance, a physiological process that in response to nutritional cues clears ~50–80% of circulating insulin, is emerging as an important factor in our understanding of the pathogenesis of type 2 diabetes mellitus (T2DM). Insulin-degrading enzyme (IDE) is a highly conserved Zn^2+^-metalloprotease that degrades insulin and several other intermediate-size peptides. Both, insulin clearance and IDE activity are reduced in diabetic patients, albeit the cause-effect relationship in humans remains unproven. Because historically IDE has been proposed as the main enzyme involved in insulin degradation, efforts in the development of IDE inhibitors as therapeutics in diabetic patients has attracted attention during the last decades. In this review, we retrace the path from Mirsky’s seminal discovery of IDE to the present, highlighting the pros and cons of the development of IDE inhibitors as a pharmacological approach to treating diabetic patients.

## 1. Introduction

Insulin-degrading enzyme (IDE) is a neutral Zn^2+^-metallo-endopeptidase that is ubiquitously expressed in insulin-responsive and -non-responsive cells [1]. IDE is evolutionarily ancient, with homologs present in phylogenetically diverse organisms of every kingdom [2]. As its name implies, IDE has a high affinity for insulin, but it can degrade a wide range of other peptide substrates, including glucagon, β-amyloid, and chemokine ligand 3 [1,3].

The subcellular localization of IDE is primarily cytosolic, but it is also present in peroxisomes, mitochondria, and endosomes [1]. Membrane-associated forms of IDE have been reported [4,5], but the nature of the attachment to membranes remains undefined. IDE has also been shown to be secreted from a number of cell types [6,7,8], in some cases in a manner dependent on differentiation state [9]; however, IDE lacks a conventional signal peptide and is not exported via the classical secretion pathway, so precisely how and to what extent it gains access to the extracellular space remains controversial [7,9,10].

### 1.1. Molecular, Structural, and Cellular Biology of IDE

The *Ide* gene is located on human chromosome 10 q23-q25 and is synthesized as a single polypeptide with a molecular weight of ~110-kDa [11,12]. IDE expression is regulated during cell differentiation and growth in rat muscle, lungs, brain, testis, uterus, tongue, skin, spleen, and thymus [13,14]. In rat liver, the activity of IDE decreases with aging [15].

The open reading frame of human, rat, and mouse *Ide* RNA contains two possible translation initiation sites: Met^1^-IDE and Met^42^-IDE. The shorter isoform (Met^42^-IDE) is the canonical and predominant isoform expressed in tissues and cells [16]. The Met^1^-IDE isoform is predicted to be less efficiently translated, resulting in the addition of 41-amino-acid N-terminal mitochondrial targeting sequence [16]. Additionally, a splice isoform in which exon 15a is replaced by a novel exon 15b has been identified [17]. This isoform is present in both cytosol and mitochondria. The 15b-IDE isoform can exist as homodimer or as heterodimer with the 15a isoform, and its catalytic efficiency against insulin is lower than the canonical 15a-IDE isoform [17].

IDE assembles as a stable homodimer where each monomer is comprised four homologous domains: The first two domains constitute the N-terminal portion (IDE-N), and the last two the C-terminal portion (IDE-C), and IDE-N and IDE-C are joined by an extended loop of 28 amino acids [18,19,20]. In addition, IDE can exist as an equilibrium of monomers, dimers, and tetramers [21]. The active site of IDE is located inside domain 1 and consists of a catalytic tetramer (HxxEHx_76_E), in which two histidine residues (H108 and H112) and a glutamate (E189) coordinate the binding of the Zn^2+^ ion and a second glutamate (E111) plays an essential role in catalysis. Although the catalytic site is within IDE-N domain, IDE-C is necessary for correct substrate recognition [18]. The overall structure of IDE resembles a clamshell, with IDE-C and IDE-N comprising bowl-shaped domains with their interiors facing one another, connected by a hinge, and together forming an internal chamber. These domains can pivot on the hinge, thus adopting “open” and “closed” conformations [18]. Additionally, there is extensive hydrogen bonding between the two halves of IDE, creating a “latch” that tends to maintain the protease in the closed conformation [18,22]. To facilitate binding and subsequent cleavages at the catalytic site, larger substrates interact with an exosite within domain 2 located ~30 Å away from the active-site Zn^2+^, which anchors the N-terminus of several substrates [1,18].

### 1.2. Historical Role of IDE in Hepatic Insulin Clearance

The existence of a proteolytic activity in rat tissue extracts that inactivated insulin was first described by Mirsky and Broh-Kahn in 1949 [23]. The enzymatic activity was a mixture of specific and non-specific proteases dubbed insulinase. Partially purified IDE from rat skeletal muscle revealed that the protease was specific for insulin but not proinsulin [24,25]. Using human erythrocytes, Shii and colleagues purified IDE to homogeneity, showing that its activity was inhibited by both sulfhydryl-modifying reagents and chelating agents [26]. Further characterization of extracts from human erythrocytes, and rat muscle, liver, kidney, or brain tissues showed that (^125^I)-insulin can be cross-linked to cytosolic IDE [27].

In cell-free systems, with the aid of labelled (^125^I)-insulin isomers and purified IDE from *Drosophila* [28], rat skeletal muscles [28,29,30,31,32,33], and human fibroblasts [34] it was shown that IDE cleaves two peptide bonds in the A chain of intact insulin and seven peptide bonds in the B chain. The A chain cleavages occur at the A13-A14 and A14-A15 peptide bonds. The major B chain cleavage sites take place at the B9-B10, B13-B14, B16-B17, and B25-B26 peptide bonds, and the minor sites at the B10-B11, B14-B15, and B24-B25 peptide bonds. Duckworth and colleagues postulated that cleavage at the B16-B17 peptide bond may alter insulin action, since the tyrosine residue at position B16 is involved in binding of the hormone to its receptor [31].

The above-mentioned studies showed that IDE can degrade insulin in vitro, but importantly, early observations made by Terris and Steiner [35], later confirmed by Duckworth and colleagues [36], demonstrated that isolated rat hepatocytes in primary cultures, with essentially no degrading activity present in the medium, were able to degrade insulin. Of note, the degradation of insulin by hepatocytes yielded identical products to those generated by purified IDE in vitro [36,37,38]. Furthermore, insulin internalization and degradation by hepatocytes was susceptible to inhibitors such as N-ethylmaleimide [36], bacitracin [39,40,41], and monoclonal antibodies [42] that disrupt IDE activity.

Although these and other results suggest that IDE plays a role in the intracellular processing of insulin, it remains the case that IDE is primarily localized within the cytosol and also lacks a signal peptide. How, then, could internalized insulin interact with the enzyme in vivo? The role of IDE as an intracellular protease of insulin in hepatocytes was reinforced by Shii and colleagues by showing that the enzyme can be cross-linked to (^125^I)-insulin in intact HepG2 cells [43]. As they showed before, IDE not only can interact with insulin in vitro [27], but also in intact cells, supporting a role of the protease in the in vivo processing of insulin. This study also indicated that (^125^I)-insulin must first interact with the insulin receptor before it comes in contact with IDE, and that the hormone must be internalized before IDE comes in contact with (^125^I)-insulin. Almost 90% of the labelled IDE was found in the cytosolic fraction, although the precise site for the degradation of insulin was not identified in the study.

In the liver, insulin binding to its receptor is the initial [35] and the rate-limiting step for insulin internalization and degradation [44,45,46]. To begin characterizing the mechanisms and subcellular compartments relevant to insulin metabolism, Duckworth and colleagues examined the effect of chloroquine (an inhibitor that prevents acidification of the endosome, resulting in accumulation of intracellular vesicles containing insulin [47]) and dansylcadaverine (an inhibitor that blocks receptor-mediated endocytosis [48]) on insulin degradation. Results from Duckworth and colleagues were interpreted as revealing the existence of intracellular and extracellular pathways for insulin degradation within isolated hepatocytes [36,49]. In addition, these studies shed light on the apparently discordant results among various studies investigating the effect of IDE inhibitors on cellular insulin degradation.

To further elucidate the site of the initial degradation step of insulin in vivo, different groups examined insulin degradation by the liver by injecting rats with radiolabeled insulin, then isolating the insulin degradation products from endosomes. These studies showed that substantial insulin degradation occurs in hepatic endosomes and allowed the identification of endosome-associated degradation products. These degradation products have intact A chains with cleavages in the B chain of insulin at B16-B17, B24-B25, and B25-B26 peptide bonds [50,51,52]. Many of the primary sites of cleavage of internalized insulin are consistent with those produced by purified IDE, suggesting that IDE mediates endosomal degradation of insulin. However, as we discuss in Section 3, the role of IDE in endosomal proteolysis of internalized insulin remains controversial, even though many of the primary sites of cleavage of internalized insulin are consistent with those produced by purified IDE [53,54,55].

In summary, the work conducted at different laboratories led to the proposal of a two-part model for hepatic insulin metabolism. In the extracellular pathway, uptake and initial degradation of insulin is dependent on the hormone first binding to its receptor [35,46], where a membrane-associated process results in partial degradation of some of the receptor-bound insulin and release of the degradation products from the cell into the medium [46,56,57]. This extracellular pathway does not require internalization of insulin [56,58], and the degradation products are postulated to result from insulin degradation on the membrane by IDE [49,59,60]. It has been estimated that about half of the insulin degraded by cultured hepatocytes is due to membrane-associated degradation [36,46]. In the intracellular pathway, some of the receptor-bound insulin is shunted to the plasma membrane and released intact [61], but the remainder of the receptor-bound insulin, representing the vast majority, is trafficked to the endolysosomal system for intracellular degradation [62,63]. Both processes involve clustering of the receptor-insulin complexes, invagination of the membrane into coated pits, and pinching off of the pits from plasma membranes to form an endosome [54,64]. Additionally, insulin can be internalized via non-coated pits and may have different intracellular pathways for its degradation [65]. The interior of endosomes rapidly acidifies due to proton pumps resulting in the dissociation of the insulin-receptor complexes, facilitating the process of degradation of free dissociated insulin by endosomal acid proteases, such as cathepsin D [54,66,67,68]. Because IDE is a neutral peptidase, and because cellular acidosis also inactivates IDE by modulating its oligomerization state [69], it has been proposed that IDE initiates degradation of insulin in the neutral environment of early endosomes while the hormone is still bound to its receptor [70,71], with subsequent degradative steps of internalized insulin occurring in the acidic environment of late endosomes.

### 1.3. Role of IDE in Insulin Clearance in Diabetes and Obesity

Over the past several decades, numerous studies have identified type 2 diabetes mellitus (T2DM) [72,73,74,75,76,77,78,79,80] and obesity [81,82,83,84,85,86,87,88,89,90,91,92] as determinant factors associated with impaired insulin clearance. Other metabolic abnormalities, such as non-alcoholic steatohepatitis [77,92,93,94,95], hepatic diseases [96,97,98,99], polycystic ovarian syndrome [100], and metabolic syndrome [101,102], as well as aging [78,103,104] and ethnicity [105,106,107,108,109,110,111], have also been linked to reduced insulin clearance.

Interestingly, genetic polymorphisms within or near the *Ide* locus have been linked to increased risk for T2DM in different ethnicities [112,113,114,115,116,117,118,119,120,121,122,123,124,125,126]. Likewise, *Ide* polymorphisms have been associated with obesity [123], metabolic syndrome [127], polycystic ovary syndrome [128], and decreased hepatic insulin clearance [121]. Finally, *Ide* coding mutations have been associated with the development of T2DM in the Goto-Kakizaki rat model [129,130].

Some studies have shown an association between reduced IDE activity or expression levels and altered insulin metabolism. Thus, Fosam and colleagues showed that in African Americans, who are at a higher risk for developing T2DM compared with non-Hispanic whites, lower IDE activity in the liver was associated with reduced insulin clearance and higher plasma insulin levels [111]. Sofer and colleagues found higher serum IDE levels in subjects with metabolic syndrome compared to control subjects [131]. Additionally, this group showed a direct correlation between circulating IDE levels and triglycerides, insulin, and C-peptide; whereas HDL-cholesterol was inversely associated [131]. Pivovarova and colleagues, using gene expression profiling by microarrays, showed decreased hepatic *Ide* expression in subjects with T2DM [132]. In another intriguing finding, Fawcett and colleagues, using adipocytes isolated from fat deposits obtained from subjects undergoing elective abdominal surgery, showed that insulin degradation, potentially due to IDE, was lower in visceral fat from diabetic patients than from non-diabetic subjects [133]. Interestingly, short-term (3-day) feeding of a high carbohydrate/low fat diet, independent of total energy intake, markedly reduced insulin clearance in healthy non-obese subjects. In contrast, 3 days of a high fat/low carbohydrate diet resulted in an increase in insulin clearance [134]. Although the cause-effect relationship between IDE and impaired insulin clearance in the setting of obesity has been extensively investigated in rodents, a recent review of the literature revealed that no conclusive information could be drawn about the impact of obesity on hepatic IDE levels and activity due to the use of different experimental models and the varying lengths and compositions of the dietary treatments [135].

### 1.4. Historical Interest in IDE as a Pharmacological Target in Hepatic Insulin Clearance

Mirsky and colleagues were the first to describe the existence of an inhibitor of IDE in liver extracts [136,137,138]. Of note, the intravenous injection of rat liver extract into rabbits resulted in an increased insulin sensitivity and decreased in fasting blood sugar [139]. In addition, Mirsky and colleagues reported that sulfonylurea-mediated inhibition of IDE in the liver was associated with the hypoglycemic effect of these drugs [140,141]. These early findings helped shape the idea that pharmacological inhibition of IDE may be beneficial by decreasing insulin clearance and thereby increasing endogenous insulin availability, leading to reduced blood glucose levels in T2DM patients. Recently, we reported that IDE protein levels are higher in T2DM patients treated with insulin than in those treated with oral hypoglycemic drugs [142]. On the other hand, Standl and colleagues showed that sulfonylureas treatment was associated with an increase in IDE activity in erythrocytes of T2DM patients and in patients with secondary failure of oral therapy, but was not altered in well-controlled type 1 diabetic patients treated with insulin [143]. Finally, the route of insulin administration was found to affect IDE activity in diabetic patients, with subcutaneous but not intravenous injections being associated with changes in the activity of the enzyme [144].

## 2. IDE Inhibitors and Insulin Clearance

Given IDE’s role in degrading insulin, and given insulin’s central role in T2DM, it is no surprise that there has been considerable interest in IDE inhibitors since its discovery in 1949 [23]. Indeed, the discoverers of IDE, Mirsky and colleagues, almost immediately went on to describe endogenous inhibitors of IDE present in rat tissue extracts [138]. As was true for the partially purified insulin-degrading protease activity they dubbed insulinase, however, the exact identity of these inhibitors remained obscure. Nevertheless, Mirsky and colleagues utilized these and other functionally characterized IDE inhibitors to test the idea that inhibition of IDE might prove to be an alternative way to treat diabetes—namely, by preventing the breakdown of endogenous insulin rather than supplementing with injection of exogenous insulin. Publishing in *Science* in 1955, Mirsky and Perisutti reported that a non-proteinaceous insulinase inhibitor purified from beef liver potentiated the hypoglycemic action of insulin in both rats and rabbits [145]. These and other results fueled strong interest in the development of IDE inhibitors as therapeutics. Reflecting the strong interest in this idea at the time, several antidiabetic drugs emerging in the late 1950s were initially believed to act by inhibiting IDE [146,147,148].

Prior to the development of selective IDE inhibitors, several studies utilized non-specific inhibitors of IDE—such as zinc-chelators, thiol-alkylating compounds, and the cyclic peptide bacitracin—in attempts to assess the functional role of IDE in insulin catabolism. Many of these studies supported the idea that IDE degrades insulin intracellularly—even within the cytosol. For instance, non-specific inhibitors of IDE decreased intracellular insulin degradation in intact HepG2 cells [149], rat L6 myoblasts [150], and mouse BC3H1 muscle cells [151]. Similarly, neutralizing monoclonal antibodies against IDE almost completely abolished the insulin-degrading activity of IDE from erythrocytes, and microinjection of these antibodies into HepG2 cells reduced intracellular (^125^I)-insulin degradation by ~50% [42]. Intriguingly, a sizable body of work suggests that insulin is trafficked to the nucleus, with cytosolic IDE being a barrier to this translocation [152]. For instance, treatment of H35 rat hepatoma cells with the broad-spectrum zinc chelator, 1,10-phenathroline, reduced insulin degradation and led to increased accumulation of (^125^I)-insulin in the nucleus, together with increased association of (^125^I)-insulin specifically with cytosolic IDE [153,154]. Finally, non-specific inhibitors of IDE were found to increase transepithelial transport of insulin through the small intestine [155] and ileum [156] in rats. While these studies are indeed intriguing, they must be viewed as only suggestive in nature, owing to the non-specific nature of the inhibitors employed (with the exception of studies utilizing neutralizing monoclonal antibodies). These topics would be worth revisiting with more selective IDE inhibitors.

Despite growing evidence of the physiological and pathophysiological importance of IDE, truly selective and potent small-molecule inhibitors of the protease did not emerge until 2010 [157], over 60 years after the discovery of IDE [23]. Several high-throughput screening (HTS) campaigns had in fact been conducted in years prior, but they proved largely unsuccessful (and unpublished) for a variety of reasons. For example, Leissring and colleagues screened a library of ~32,000 compounds comprised of 704 FDA-approved compounds, 352 natural products, a tetrapeptide library consisting of all possible permutations of 8 amino acids (Glu, His, Lys, Pro, Gln, Val, Trp, Tyr), and an additional 27,300 small-molecule pharmacophores conforming to Lipinski’s rules [158]. This campaign was conducted using a fluorogenic peptide substrate, in the presence and absence of ATP, with the goal of identifying IDE activators that disrupt the interaction of IDE with ATP, which had been reported to be an inhibitor of insulin degradation [159]. This screen was successful in identifying a few modest inhibitors (as well as activators) of IDE, albeit with two key caveats. First, many compounds were thiol-alkylating compounds, which tend to be non-specific. Second, and quite important, compounds that inhibited (or activated) the degradation of short fluorogenic compounds were shown to be inactive or even to have opposite effects when tested using full-size physiological substrates such as Aβ or insulin [158]. Results such as these reinforced the fact that IDE exhibits highly substrate-selective enzymological properties, making it critical that screening campaigns be conducted with the substrate of interest, rather than with a more convenient or less expensive surrogate, such as a fluorogenic peptide. This fact prompted the development by the Leissring group of several HTS-compatible assays for various IDE substrates, including Aβ [160], glucagon [161], and amylin [162].

HTS campaigns conducted with full-length endogenous IDE substrates, unfortunately, also proved largely unfruitful. For instance, Leissring and colleagues used their newly developed Aβ degradation assay [160] to search for activators of IDE, in this case pursuing compounds that disrupt the binding and inhibition of IDE by fatty acids [163], specifically by linoleoyl-coenzyme A [164]. Although optimized to detect both activators and inhibitors of IDE, this screen of ~100,000 small molecules yielded no inhibitors other than thiol-alkylating compounds. Several additional unpublished HTS campaigns, on a total of more than 2.4 million compounds, in both cell-based and in vitro screens, yielded similar results (M.A.L. unpublished observations). This lack of success of identifying compounds via HTS demonstrates that IDE is a particularly difficult drug target, helping to explain why IDE inhibitors emerged so late after its discovery.

**Figure 1:**
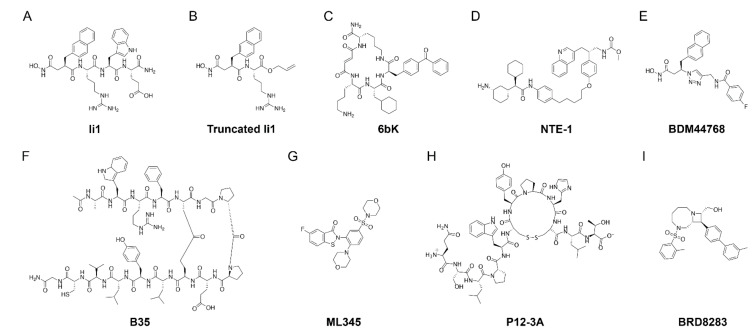
Structures of and common names for insulin-degrading enzyme (IDE) inhibitors.

**Figure 2:**
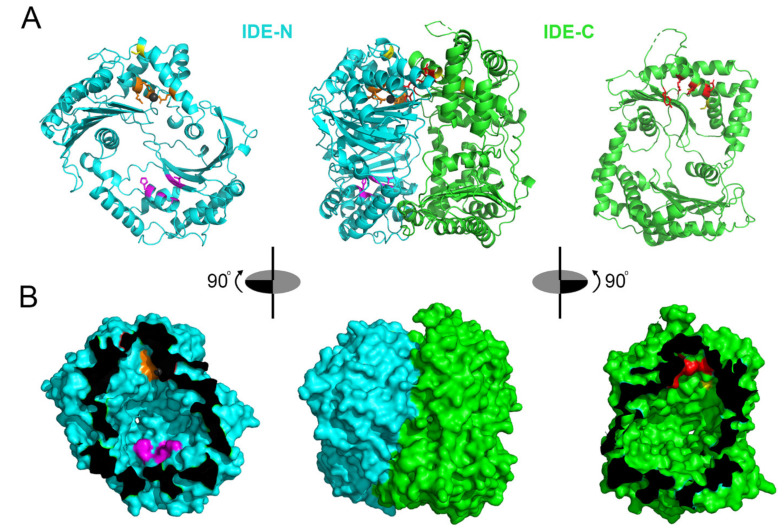
Structure of human IDE and regions targeted by different inhibitors. (**A**,**B**), Illustration of a single monomer of IDE (center) and IDE-N (left, cyan) and IDE-C (right, green) depicted in ribbon (**A**) and surface (**B**) representations. Zinc-binding and catalytic residues within the active site of IDE-N are depicted in orange, with the zinc atom shown as a gray sphere. Residues within IDE-C that make up the second portion of IDE’s bipartite active site are shown in red. Cysteine residues targeted by thiol-modifying inhibitors are shown in yellow. The distal exosite is shown in magenta. In (**B**), note that the portions of IDE-N and IDE-C that are adjacent when the protease is in the closed conformation are depicted in black. Figures generated in Pymol [169] from PDB 2G54 [20].

As the preceding makes clear, a breakthrough in IDE inhibitor development would require an alternative strategy. Leissring and colleagues elected to use a rational-design approach to develop peptidic, zinc-targeting compounds optimized for IDE [157]. To that end, they first determined the cleavage-site specificity of IDE de novo using a proteomic method developed by Turk and colleagues [165]. This analysis revealed that IDE has a preference for Tyr and Phe at the P_1′_ position, and Arg at the P_2′_ position, with lower preferences at the remaining positions. A conventional hydroxamic acid (Hx) comprising the sequence Hx-Phe-Arg-Trp-Glu yielded a K_i_ value of ~110 nM. The team then optimized the original compound by synthesizing a focused library of retro-inverso peptide hydroxamates with different unnatural amino acids at the P_1′_ position, obtaining a 100-fold improvement when the Phe was substituted with 2-naphthylalanine (2-Nap). Synthesis of a conventional hydroxamic acid with 2-Nap at the P_1′_ position yielded a compound, dubbed Ii1 (IDE inhibitor 1; Figure 1A and Figure 2), with a K_i_ value of ~1 nM against multiple substrates. Significantly, despite being a relatively crude, first-generation peptide hydroxamate, **Ii1** proved to be surprisingly selective for IDE vis-à-vis many [157] (but not all [166]) conventional zinc-metalloproteases, likely reflecting the evolutionary and structural divergence of the inverzincin superfamily [167]. Notably, the crystal structure of the IDE-**Ii1** complex revealed that Ii1 interacts with multiple residues in both IDE-N and IDE-C, suggesting that Ii1 might inhibit IDE, in part, by maintaining it in the closed conformation.

Although potent and reasonably selective, Ii1 had the disadvantage of being relatively large as well as surprisingly difficult to synthesize. Towards the goal of optimizing Ii1, Leissring and colleagues developed a series of truncated variants, including a 455-Da allyl ester comprising only the 2-Nap and Arg residues (Figure 1B and Figure 2) [168]. Unexpectedly, these variants showed remarkable substrate selectivity, differing in potency by as much as 300-fold for different substrates, despite being active-site-directed compounds [168]. This finding suggests that, in at least some cases, the inhibition of IDE may depend on tertiary interactions among the enzyme, substrate and inhibitor, lending support to the possibility of developing substrate-selective compounds.

The development of the first in vivo-compatible IDE inhibitor—a particularly selective one—was achieved by Liu and colleagues using another highly novel approach. This team first synthesized a library of cyclic peptides containing conventional and non-conventional amino acids, each tagged with a unique DNA-based “bar code” [166]. Compounds selected for the ability to bind to immobilized IDE were identified by sequencing, then the top compounds were optimized and characterized extensively, using crystallography and site-directed mutagenesis. This effort led to the development of 6bK (Figure 1C and Figure 2), a reasonably potent IDE inhibitor (IC_50_ = ~100 nM against insulin) that proved to be exceptionally selective because it targeted the wholly unique exosite within IDE rather than its highly conserved active site [166]. As a result, the mechanism of action of 6bK was steric blockade of substrates accessing the internal chamber of IDE. Liu and colleagues subsequently refined this DNA-templated approach to develop other potent IDE inhibitors with more drug-like properties [170,171]. When tested in mice fed a HFD, 6bK improved oral glucose tolerance and insulin tolerance in normal and diabetic mice, with no effect evident in IDE-KO mice [166], consistent with the long-predicted outcome. Surprisingly, however, 6bK dramatically worsened intraperitoneal (IP) glucose tolerance 1 h after administration [166], a result that was hypothesized to be due to the “incretin effect” involving hormones other than insulin [172]. In agreement with this, 6bK treatment produced increases not only in plasma insulin, but also in amylin and glucagon, each with unique temporal profiles [166].

The next in vivo-compatible IDE inhibitor was developed by Sloop and colleagues at Eli Lilly. Once again, a novel approach was required to develop a potent and selective inhibitor, in this case, fragment-based drug design [173]. Briefly, compound screening was used to identify two small molecules that bind to IDE at different exosites within the internal chamber. These fragments were subsequently joined with an appropriately sized linker, resulting in “dual-exosite” inhibitors dubbed NTE-1 (Figure 1D and Figure 2) and NTE-2 [173]. Similar to 6bK, administration of NTE-1 to diet-induced obese mice improved the glucose excursion in oral glucose tolerance tests [173]. However, this team found no effect on insulin tolerance, despite an increase in plasma insulin levels post-glucose challenge, furthermore, euglycemic clamping studies revealed no changes in insulin responsiveness [173]. By contrast, however, plasma amylin levels were increased by NTE-1 treatment [173].

Duprez-Poulain and colleagues developed yet another family of IDE inhibitors comprising novel variants of Ii1 [174]. This team developed a 2-Nap-containing hydroxamate warhead containing an azide moiety, permitting it to be readily attached to a variety of alkynes via a “click-chemistry” reaction, thereby generating a series of hydroxamates containing 1,4 or 1,5- disubstituted triazoles [175]. Relying on the fact that click chemistry can proceed under neutral, aqueous conditions [176], the team used a clever approach called kinetic target-guided synthesis [177] to generate optimized variants: They exposed the Hx-2-Nap warhead to a mixture of alkynes in the presence of IDE, thus favoring the coupling of the warhead to specific alkynes predisposed to bind favorably to the active site of IDE. The resulting compounds, including BDM44768 (Figure 1E and Figure 2) and BDM44619, exhibited K_i_ values as low as ~60 nM [174]. As was true for Ii1, crystal structures of these compounds complexed to IDE revealed significant interactions with both IDE-N and IDE-C, reinforcing the idea that the mechanism of inhibition may involve maintaining IDE in the closed conformation. Consistent with this, in the presence of BDM44768, IDE was found to adopt a light-scattering profile similar to that predicted for the closed conformation of the protease [174]. Treatment of mice with BDM44768 resulted in modestly improved insulin tolerance along with modestly increased plasma insulin levels after IP insulin administration [174]. However, in striking contrast to the results obtained with 6bK and NTE-1, BDM44768 administration resulted in a significant worsening of oral and IP glucose tolerance in both wildtype B6 mice and non-obese diabetic (NOD) mice. No effect was observed in the latter mouse lines lacking IDE, implying that the effect was in fact dependent upon IDE [174]. Of special interest to the topic of insulin clearance, BDM44768 treatment increased circulating insulin levels after glucose challenge in NOD but not wildtype mice [174]. Finally, this team established that BDM44768 did not alter hepatic gluconeogenesis as assessed by pyruvate tolerance testing [174].

Li and colleagues employed a structure-based rational design approach, based on the IDE-insulin B chain co-crystal structure [20], to devise a stabilized β-hairpin peptide that mimics the insulin B chain binding sequence (EALYLVCG) [178]. The optimized inhibitor, B35 (Figure 1F and Figure 2), efficiently inhibited IDE activity (IC_50_ = ~22 nM) and showed ≥1000-fold selectivity for inhibition of IDE vis-à-vis other metalloproteases, such as angiotensin-converting enzyme, endothelin-converting enzyme-1, and neprilysin [178]. B35 administration resulted in improved oral glucose tolerance in diet-induced obese mice, but no effect was observed in both glucose and insulin tolerance in lean mice [178].

Using a cell-based ultra-HTS format and follow-up optimization [179], the Leissring team developed, ML345 (Figure 1G and Figure 2), a compound with a particularly interesting “quasi-irreversible” property: ML345 is a thiol-modifying compound, but it forms a disulfide bond that can be broken in a reduced environment [180]. By virtue of this unique property, ML345 can selectively target extracellular IDE, which is present in an oxidized environment, while sparing intracellular IDE within the reduced environment of the cytosol, making it an important experimental probe for disentangling the relative contributions of different pools of IDE to insulin degradation and other processes. More recently, the Leissring team used the classic technique of phage display to identify peptidic inhibitors of IDE intended for topical applications [181]. The rational here was to promote wound healing by blocking insulin degradation by IDE in wound fluid, where it is abundant [182,183]. In this case, the objective was not to identify highly potent compounds, but instead to develop IDE inhibitors comprised solely of natural amino acids so that they would be both non-toxic and inexpensive to generate. Consistent with well-established effects of insulin in wound healing [184], the peptidic IDE inhibitor P12-3A (Figure 1H and Figure 2) was found to block extracellular insulin degradation and promote collagen production in fibroblasts, as well as to potentiate the migration of keratinocytes in a scratch wound migration assay [181]. This study highlights the fact that insulin catabolism by IDE plays an important role in processes other than blood sugar regulation.

The Liu team recently accomplished a remarkable, crowning achievement: The development of an insulin-specific IDE inhibitor; that is, an inhibitor that exclusively blocks insulin degradation, while leaving intact the ability of IDE to degrade other substrates [185]. To achieve this, Liu and colleagues employed a fluoresceinated version of 6bK, using fluorescence polarization to identify compounds that displace the fluorescently tagged probe from IDE’s exosite [185]. Subsequent optimization of identified hits was conducted by screening derivatives against multiple IDE substrates. This led to the compound BRD8283 (Figure 1I and Figure 2), which inhibits insulin degradation reasonably potently (IC_50_ = ~100 nM) and, most remarkably, selectively targets insulin degradation by IDE [185]. As crystallographic analysis revealed, the ability of BRD8283 to selectively target insulin is due to selective steric hindrance: The compound binds to a region of IDE that is uniquely occupied by insulin—a particularly bulky and inflexible substrate—and not by other substrates. If this compound, or subsequent derivatives, can be utilized in vivo, this unique property will be extremely helpful for disentangling IDE’s role in blood sugar regulation.

## 3. Activation vs. Inhibition as a Therapeutic Strategy

Although IDE’s role as a protease of insulin has been extensively investigated, there remains considerable doubt that it plays a significant role in hepatic insulin clearance in vivo. Thus, as seen in Table 1, effects of IDE inhibitors on insulin clearance in vivo have not been assessed or resulted in no effects, such as the NTE-1 inhibitor.

The main lines of evidence implicating IDE in hepatic endosomal proteolysis of insulin are: First, its high affinity for insulin, suggesting that the enzyme is very specific for the hormone; second, the finding that cleavage insulin products obtained from isolated hepatic endosomes are similar to those produced by purified IDE; and third, the detection of small pools of IDE in endosomes. However, its role in endosomal degradation of insulin in vivo remains controversial. The work claiming to localize IDE within endosomes from rat livers [70] and kidneys [186] utilized outdated methodology, and there was considerable potential for cross-contamination by IDE within other organelles such as mitochondria. Likewise, more recent work by Song and colleagues postulating that IDE might be recruited to endosomes via its polyanion-binding site fails to show definitively that IDE is, in fact, present in endosomes rather than within or merely associated with other membranous organelles [187].

Another reason to doubt the idea that IDE processes insulin within endosomes is the fact that IDE activity is modulated by pH and would therefore be inactive in the acidic environment of late endosomes. It has been proposed that IDE initiates degradation of insulin in the relatively neutral environment of early endosomes while the hormone is still bound to its receptor [70,71]. Presuming that IDE can gain access into early endosomes, despite the lack of a signal peptide sequence, the relative contribution of this step to the overall process of insulin degradation remains questionable. Moreover, the fact that IDE must completely encapsulate its substrates to process them [20] casts considerable doubts on the idea that IDE could degrade insulin bound to its receptor [71]. These considerations suggest that pharmacological inhibition of IDE, whatever other effects it might have in vivo, would be expected to have little impact on hepatic insulin clearance per se.

In studies that have implicated IDE in hepatic insulin clearance, approximately half was found to be degraded on the cell membrane, without requiring internalization of insulin in cultured hepatocytes [36,46,49,56,58,59,60]. Although this extracellular pathway appears to be mediated by IDE, the details of this process are poorly understood (e.g., whether IDE is localized on the outer or the inner side of the plasma membrane), and its relevance for hepatic insulin clearance in vivo has not been established. In light of this uncertainty, it may be that the highly varied efficacy of different IDE inhibitors on hepatic insulin clearance tested in mice is related to their ability access IDE within relevant compartments. Further work is necessary to understand the extent to which plasma membrane-associated degradation of insulin is mediated by IDE and whether IDE inhibitors can affect this process.

Other minor concerns have been raised about a role in endosomal proteolysis of internalized insulin. For example, IDE can degrade a large number of substrates, including some with stronger affinities than insulin [188], suggesting it may have other roles besides insulin catabolism. Also, the presence of IDE in both insulin-responsive and non-responsive cells, as well as its regulation during liver development, also suggests other functions besides insulin degradation [13,14,15,189].

In light of these considerations, how can we explain the extensive evidence in the literature indicating that increasing IDE activity increases cellular insulin degradation, and conversely, that decreasing its activity reduces insulin degradation? [1]. First, most of these studies were performed not with hepatocytes, but with various other cell types, instead. Second, NTE-2 had little impact on insulin clearance in HEK cells [173]. Third, overexpression of IDE has not uniformly been found to increase the rate of insulin degradation in cells [11,190]. Finally, these in vitro data may not parallel the impact on hepatic insulin clearance in vivo.

The impact of IDE on insulin proteostasis in vivo is similarly controversial. Thus, pancellular deletion of *Ide* in mice resulted in significant increases in plasma insulin in some studies [191,192] but no changes in others [193,194]. Moreover, we have shown that mice with liver-specific deletion of *Ide* (L-IDE-KO mice) display normal hepatic insulin clearance when fed a regular or high-fat diet (HFD) [195,196]; and that hepatic overexpression of IDE in mice fed HFD resulted in unaltered clearance, despite lower plasma insulin levels [196]. These findings have cast doubt on idea that IDE is an endosomal protease of insulin, leading Najjar and Perdomo to propose a new mechanistic model for hepatic insulin clearance, in which the coordinate functions of IDE and carcinoembryonic antigen-related cell adhesion molecule 1 (CEACAM1) regulate insulin action and disposal in the liver [135]. In this model, IDE’s main effect on insulin catabolism appears to involve modulating intracellular trafficking of the insulin-insulin receptor complex, particularly, on insulin receptor recycling to plasma membrane [135].

Additional studies by our group in mice with tissue-specific deletion of IDE have revealed an unrecognized role for IDE in regulating hepatic insulin sensitivity, resulting in lower insulin sensitivity when IDE activity is reduced [195,196]. These results contrast with early works that prompted the notion that inhibition of cellular degradation of insulin by IDE would potentiate and prolong the effect of insulin in vivo [197,198,199]. In marked contrast to previous thinking, we found that overexpression of IDE in liver resulted in improved glucose homeostasis in diet-induced mice [196], suggesting that the development of IDE activators, rather than IDE inhibitors, may be a viable pharmacological approach for the treatment of diabetic patients. Further work is needed to follow this less explored facet of IDE biology.

As discussed, several IDE inhibitors, including 6bK, NTE-1, and B35, have been shown to improve glucose and/or insulin tolerance in mice [166,173,178]. However, it is critical to recognize that the effects of these inhibitors on hepatic insulin clearance have not been directly assessed (except in the case of NTE-1, which was found to have no effect on insulin clearance [173]). This is a crucial issue when testing IDE inhibitors in vivo, since off-target effects on insulin secretion and peripheral insulin sensitivity might be responsible for improving (or worsening) glucose metabolism. Thus, future studies using IDE inhibitors should investigate potential effects on insulin secretion and clearance, in addition to glucose and insulin tolerance. Likewise, in view of the multi-substrate activity of IDE, circulating levels of glucagon and amylin, at minimum, should be tracked, in addition to insulin. At the same time, it is important to establish optimal conditions for administering IDE inhibitors (i.e., transient vs. chronic, and the appropriate time of the intervention along the progression of the disease) and—importantly—to assess whether individual inhibitors can penetrate cells or not. Longitudinal studies in diabetic mouse models should also be considered. Finally, future studies should take advantage of the IDE-KO and L-IDE-KO mouse models to demonstrate specificity of IDE inhibitors in vivo.

In the hypothetical case that IDE inhibition does impact circulating insulin levels, another facet relevant to the potential use of IDE inhibitors in clinical settings is the impact on hyperinsulinemia. While there is ample evidence that transient increases in circulating insulin levels is a physiological response (e.g., in response to a rise in glucose levels after a meal), chronic elevations might be detrimental. For instance, Mendelian randomization studies showed that individuals carrying ≥ 17 alleles that raise fasting insulin levels have an increased risk of elevated blood pressure, cardiovascular disease, and T2DM [200]. Following this line of thinking, Bergman and colleagues have hypothesized that reduced hepatic insulin degradation is a cause rather than a result of insulin resistance [201]. Reduced insulin clearance by liver would cause hyperinsulinemia, resulting in peripheral insulin resistance by overexposure to endogenous insulin. From this point of view, inhibition of hepatic insulin clearance may not be an appropriate pharmacological approach for treating diabetic patients.

Contrarily, Gastaldelli and colleagues reported that the development of insulin resistance in obese subjects was associated with decline in hepatic insulin clearance [202], but they hypothesized that reduced clearance would be an important mechanism that contributes to the compensatory hyperinsulinemia in an attempt to maintain normal glucose homeostasis in diabetic patients. This argument favors the rationale of using IDE inhibitors to inhibit hepatic clearance and increase circulating insulin in the late phases of T2DM.

Interestingly, Kim and colleagues showed that treatment with salsalate in non-diabetic insulin-resistant individuals improved fasting glucose and triglyceride concentration [203]. These improvements were associated with a decrease in insulin clearance rate without change in insulin secretion or action [203]. Likewise, Penesova and colleagues showed that salsalate administration in obese subjects without diabetes resulted in increased insulin levels associated with lower insulin clearance and unaltered insulin secretion [204]. Although it remains to be determined if the effect of salsalate on insulin clearance is mediated by inhibition of IDE, these studies highlight the need for the evaluation of current pharmacological interventions on hepatic insulin clearance in diabetic patients.

Although initially described as a protease of insulin, decades of work have demonstrated that IDE is a multifunctional protein with catalytic and non-catalytic functions in different tissues [1]. Thus, inhibition of IDE may cause accumulation of amyloidogenic peptides such as amylin and Aβ, increasing the risk for Alzheimer disease or other conditions in a clinical setting. Likewise, inhibition of IDE in pancreatic β-cells causes deleterious effects on insulin secretion in mice, which raises concerns of its inhibition in T2DM patients [194,205]. Thus, even if IDE inhibitors do prove to have beneficial effects on certain diabetes-related endpoints, it will be important to thoroughly investigate their effects on all organs as well as to assess their potential to influence risk for certain diseases.

Although the utility of IDE inhibitors in the treatment of diabetes awaits further investigation, their use in promoting wound healing in diabetic patients seems promising and comparatively uncomplicated. Insulin plays a critical role in wound healing, by promoting several processes, ranging from cell proliferation to the production of collagen and other extracellular matrix proteins [184]. IDE is abundant in wound fluid [183], where it is capable of degrading insulin [182]. Notably, Duckworth and colleagues reported that diabetic patients with worse outcomes (i.e., amputation of extremities) had higher wound fluid insulin degradation than fluid from non-diabetic patients [183]. Conversely, Yang and colleagues showed increased expression of IDE in the skin of diabetics during wound healing, which was associated with impaired wound healing [206]. Taken together, these observations suggest that inhibition of IDE activity may be a valid therapeutic approach for promoting wound healing. The development of low-cost peptidic IDE inhibitors by Leissring and colleagues [181] will facilitate investigation of this promising area of research.

## 4. Concluding Remarks

In sum, although early studies suggested that IDE plays a key role in hepatic insulin clearance, the advent of more modern approaches, such as tissue-specific genetic ablation, has cast considerable doubt on this idea. Accordingly, the approach treating diabetes by pharmacological inhibition of IDE, predicated as it is on increasing circulating insulin levels by slowing insulin clearance, will require more thorough investigation of this topic to assess the wisdom of this approach. For now, IDE inhibitors will be instrumental as experimental tools to further elucidate the function of IDE in insulin metabolism and many other physiological and pathophysiological processes.

## Figures and Tables

**Table 1:** Effects of IDE inhibitors on insulin clearance. n.d. = not determined. **^a^** From experiments in mice, unless otherwise indicated.

Compound	K_i_/IC_50_ Value	Mechanism	Effects on Insulin Levels ^a^	Insulin Clearance ^a^	Refs.
Ii1	K_i_ = ~16 nM (insulin)	Zn-targeting and stabilizing closed conformation	Blocked degradation of extracellular insulin by CHO-IR and HeLa cells	n.d.	[168]
6bk	IC_50_ = ~100 nM (insulin)	Steric blockade of internal chamber	Elevated plasma insulin after IP insulin or glucose administration	n.d.	[166]
NTE-1	IC_50_ = ~4 nM (insulin) IC_50_ = ~3 nM (glucagon)	Steric blockade of internal chamber	No significant effect on plasma insulin levels	No effect	[173]
NTE-2	IC_50_ = ~4 nM (insulin) IC_50_ = ~150 nM (glucagon)	Steric blockade of internal chamber	No effect on extracellular insulin degradation by HEK293 cells	Little effect in HEK293 cells	[173]
BMD44768	K_i_ = ~60 nM (insulin)	Zn-targeting and stabilizing closed conformation	Elevated plasma insulin after exogenous insulin administration	n.d.	[174]
B35	IC_50_ = ~22 nM (insulin)	Active-site blockade; mimics insulin B chain sequence that binds to active site	Elevated basal plasma insulin levels Increased levels of insulin 120 min after IP injection of B35	n.d.	[178]
ML345	IC_50_ = ~23 nM (insulin)	Quasi-irreversible Cys-targeting; selectively inhibits IDE in oxidized environment	n.d.	n.d.	[180]
P12-3A	K_i_ = ~2,5 uM (insulin)	Steric blockade of active site	Blocked degradation of extracellular insulin in fibroblasts	Inhibitory effect in cultured fibroblast	[181]
BRD8283	IC_50_ = ~100 nM (insulin)	Binding to the IDE exosite region uniquely occupied by insulin	n.d.	n.d.	[185]

## Data Availability

No new data were created or analyzed in this study. Data sharing is not applicable to this article.
